# Effect of Drying Methods on Properties of Potato Flour and Noodles Made with Potato Flour

**DOI:** 10.3390/foods10051115

**Published:** 2021-05-18

**Authors:** Huan Bao, Jiaping Zhou, Jinglin Yu, Shujun Wang

**Affiliations:** 1 State Key Laboratory of Food Nutrition and Safety, Ministry of Education, Tianjin University of Science and Technology, Tianjin 300457, China; zhoujiaping@tust.edu.cn (J.Z.); yil_wsj@tust.edu.cn (J.Y.); sjwang@tust.edu.cn (S.W.); 2 School of Food Science & Engineering, Tianjin University of Science & Technology, Tianjin 300457, China

**Keywords:** potato flour, freeze drying, ethanol drying, oven drying, fresh noodles, quality characteristics

## Abstract

This work investigated the impact of three drying methods on structural and functional properties of potato flour (PF), as well as the quality characteristics of fresh noodles made from wheat-potato flours. The results indicated that ethanol drying (ED) and oven drying (OD) had small effects on the properties of starch in potato flour, however, freeze drying (FD) caused some pores and channels on the starch granules and disruption of the long- and short-range ordered structure of starch. The maximum addition of potato flour in fresh noodles was 40% for FD-PF and 50% for both ED-PF and OD-PF. With increasing addition of potato flour in noodles, the L* (lightness) values of noodles decreased gradually, while the a* (redness) and the b* (yellowness) values, as well as the hardness and springiness values of fresh potato noodles increased. This study clearly showed that drying methods have different effects on the properties of potato flour, and in turn the quality of fresh noodles made with potato flour.

## 1. Introduction

Potato (*Solanum tuberosum* L.) is the fourth-largest crop following maize, rice and wheat [1]. Starch is one of the primary components in potato tubers. Native potato starch may be a representative B-type polymorph and has low amounts of proteins and lipids. A comparatively high degree of phosphate groups bestows potato starch with unique properties, such as high pasting viscosity and swelling power [2]. Additionally, potatoes have many micronutrients, such as vitamins, minerals, phenolic compounds and a high level of dietary fiber. Additionally, potato varieties with a higher resistant starch content have potential health-promoting benefits in terms of hypoglycemic and prebiotic effects, prevention of colon cancer, and inhibition of fat accumulation [3,4], which makes potatoes a functional material in food applications. The functional and nutritional characteristics of potato flour or starch make it to be a potential substitute for cereal flour or starch to improve the textural or nutritional quality of finalized food products.

The drying methods used in common nourishment research facilities for flours or starches incorporate oven (heat) drying, freeze-drying, and ethanol (solvent-exchange) drying [5]. Previous studies reported that drying damages the surface and even the inside structures of starch granules, in the long run influencing their functional properties, such as gelatinization, retrogradation, and pasting properties; however, the drying process does not change the overall starch digestion pattern [5,6,7]. Freeze drying damages the surface and inside structure of potato starch granules. In contrast to ethanol-dehydration (room temperature) and oven drying (40 °C), freeze drying disrupted the long- and short-range molecular order of double helices in chestnut starches, and decreased swelling power and gelatinization temperatures of starches [8,9,10]. 

Preparation of commercially available potato flour usually involves boiling or steaming and heat-drying processes, which causes starch gelatinization. This treatment disrupted the structure and functional properties of potato starch in some ways, such as a decrease in the values of pasting viscosities, which may be unfavorable for the production of some steamed or frozen foods that need to be cooked before consumption [11]. To reduce the loss of the functional properties of potato starch during preparation of potato flour, a mild preparation method is necessary to ensure the contribution of potato starch to the quality of the finalized food products. However, there have been no comprehensive studies on comparing the effects of different drying methods on structural and functional properties of potato starch. Due to increasing utilization of potato flour in the food industry, there is a need to evaluate the effect of preparation methods, especially the drying process, on the functional properties of potato flour and the quality of finished food products. While drying methods can alter the structural properties of potato starch, information is scarce on the effects of drying methods on the properties of potato flour. Hence, the objective of this work is to compare the effects of different drying methods on the structure and functional properties of potato flour and the quality characteristics of the noodles made of wheat flour and potato flour. The information obtained will provide useful information for the selection of suitable drying methods for the preparation of potato flour and give indication on the application of potato flour in different food products.

## 2. Materials and Methods

### 2.1. Materials

Wheat flour was obtained from Jinshahe Group Co., Ltd. (Xingtai, China) with total starch, protein, and fat contents of 62.93, 12.87, and 1.52 g/100 g flour, respectively. The fresh potatoes were acquired from Tianjin, China. Potato tubers were stored in a refrigerator (4 °C). All the reagents were of analytical grade.

### 2.2. Preparation of Potato Flours

The fresh potatoes were washed thoroughly, peeled, and cut into small pieces with a kitchen knife and homogenized in a kitchen blender with 500 mL distilled water for 1–2 min at greatest speed. After centrifugation, the precipitates were subjected to three different drying medications described previously [5,8]. Wet PF was dried at 40 °C in an oven for 48 h, ground, and passed through a 125 μm strainer, which was referred to as PF-OD. For ethanol drying (ED), wet PF was resuspended in absolute ethanol, centrifuged, and dried at room temperature in a fume hood for 48 h, and the obtained sample was named PF-ED. For freeze drying (FD), wet PF was solidified in a −80 °C cooler and dried in a solidify dryer (LGJ-10, Four-Ring Science Instrument Plant Beijing Co. Ltd., Beijing, China), ground, and sieved through a 125 μm mesh, and the obtained sample was abbreviated as PF-FD. All the potato flour was prepared in the same conditions to ensure the accuracy of the experiment.

### 2.3. Moisture and Total Starch Content of Potato Flour

Moisture content was determined by drying flour to a steady weight at 105 °C. Total starch content of PF was determined by a Megazyme total starch (Bray County, Wicklow, Ireland) assay kit.

### 2.4. Scanning Electron Microscopy

The morphology of starch granules within the PF was imaged using a LEO 1530 scanning electron microscope (LEO, Jena, Germany). The samples were mounted on the aluminum stub using the double-sided carbon adhesive tapes and sputter-coated with gold. An accelerating voltage of 5 kV was used during imaging.

### 2.5. X-ray Diffraction (XRD)

X-ray diffraction examination was performed employing a D/max-2500 vk/pc X-ray diffractometer (Rigaku Corporation, Tokyo, Japan) working at 40 kV and 30 mA. Tests were equilibrated over an immersed sodium chloride (NaCl) arrangement at room temperature for one week before analysis. The tests were filtered from 4 °C to 35 °C, 2θ and at a checking speed of 1°/min and a step measure of 0.02°. The relative crystallinity was quantitatively assessed as a proportion of the crystalline region to the overall zone between 4 °C to 35 °C (2θ) utilizing the Origin software (Version 8.0, Microcal Inc., Northampton, MA, USA).

### 2.6. Laser Confocal Micro-Raman (LCM-Raman) Spectroscopy

The LCM-Raman spectra of the PF tests were obtained using a Renishaw Invia Raman microscope system (Renishaw, Gloucestershire, UK) prepared with a Leica microscope (Leica Biosystems, Wetzlar, Germany); a 785 nm green diode laser source was utilized. 

### 2.7. Differential Scanning Calorimetry (DSC)

The thermal properties of the PF were measured using a differential scanning calorimeter (200 F3, Netzsch, Selb, Germany) prepared with a thermal analysis data station [12]. PF (3 mg) was weighed accurately into 40 μL aluminum pans, and distilled water was added to give a water: PF ratio of 3:1 (*w*/*w*). The pans were sealed and allowed to stand overnight at room temperature before analysis, heated from 20 to 100 °C at a rate of 10 °C/min. An empty pan was used as the reference.

### 2.8. Pasting Viscosity and Gel Textural Analysis

The pasting viscosity properties of PF were determined from a Rapid Visco Analyzer (RVA-4) (Perten Instrument Australia, Macquarie Stop, NSW, Australia) concurring to STD 1 strategy. PF (2.5 g) was weighed into the RVA canisters, and the distilled water was added to make a total weight of 28 g. 

After RVA examination, the flour paste formed within the canister was fixed with parafilm to prevent moisture loss and stored at 4 °C for 10 days for textural examination. 

### 2.9. Swelling Power and Starch Solubility

The swelling power and solubility of starch were decided based on the strategy of Wang and Copeland [13], with a few adjustments as follows. Watery flour suspensions (4%, *w*/*w*) were warmed in a water shower at 92.5 °C for 30 min with normal shaking and cooled at 20 °C for 3 min and centrifuged at 13,000× *g* for 10 min. The supernatant dried at 105 °C and weighed to decide dissolvability (% of dry flour) and swelling power (g H_2_O absorbed/g dry flour).

(1) $$\mathrm{Solubility},\;S=\frac{\mathrm{weight}\;\mathrm{of}\;\mathrm{solubles}}{\mathrm{dry}\;\mathrm{weight}\;\mathrm{of}\;\mathrm{original}\;\mathrm{flour}}$$

(2) $$\mathrm{Swelling}\;\mathrm{power},\;SP=\frac{\mathrm{weight}\;\mathrm{of}\;\mathrm{swollen}\;\mathrm{granules}}{\mathrm{dry}\;\mathrm{weight}\;\mathrm{of}\;\mathrm{original}\;\mathrm{flour}}$$

### 2.10. Preparation of the Noodles Made with Potato Flour

Noodles were prepared by a noodle machine (BJM-8, Beijing, China). Potato flours were blended with wheat flour at the following weight rates of 0%, 10%, 20%, 30%, 40%, 50% (wheat flour basis, *w*/*w*). The dough was made physically by adding 50% of water and NaCl aqueous solution (final NaCl percentage: 2% flour basis) to the above flours. The dough shaped was put to rest in a plastic sack for 20 min and after that passed through a noodle machine to prepare noodles. The noodle strands of 4 mm in width were produced by the noodle machine and cooked at optimum time for further tests.

### 2.11. Color Analysis of Noodles

A chroma meter (Konica Minolta, Tokyo, Japan) was utilized to degree the color of the noodle tests. L* is a measurement of brightness (0–100), a* represents the red-green facilitates (− is green while + is red) whereas b* measures the blue-yellow arranges (− is blue with + demonstrating yellowness) of a product [14].

### 2.12. Textural Analysis (TPA) of Fresh Noodles

A standard two-cycle program was utilized to compress the gels for a 20% strain at 1.0 mm/s test speed, 1.0 mm/s pre-test speed, 1.0 mm/s post-test speed, 2 s delay time and 5 g trigger drive. Three strands of noodles were set parallel on the testing stage, and the judgments were conducted under optimal test conditions: strain, 70%; test and post-test speed, 1 mm/s; interim time, 2 s. Texture parameters of hardness, springiness, cohesiveness, gumminess, chewiness and resilience were inferred from the bends by the instrument software.

### 2.13. Statistical Analysis

All tests were performed at the slightest in triplicate and the comes about are detailed as the cruel values and standard deviations. Within the case of XRD, as it were one estimation was conducted. Investigation of change (ANOVA) by Duncan’s test (*p* < 0.05) was conducted utilizing the SPSS 10.0 Statistical Software Program (SPSS Inc., Chicago, IL, USA).

## 3. Results

### 3.1. Moisture and Total Starch Content of Potato Flour

The moisture content of PF-ED, PF-OD and PF-FD was 10.5%, 8.7% and 5.8% (Table 1), respectively. Previous research pointed out that ethanol drying and oven drying at 40 °C resulted in loss of free water, whereas freeze drying may lead to loss of bound water in starch granules [8]. The total starch contents of PF-FD, PF-OD and PF-ED were 75.4%, 73.5%, and 70.5%, respectively.

### 3.2. Granule Morphology

**Figure 1:**
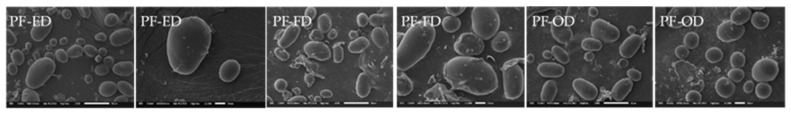
SEM images of potato flour granules subjected to different drying methods. PF-ED: potato flour dried for ethanol drying; PF-FD: potato flour dried for freeze drying; PF-OD: potato flour dried for oven drying.

Scanning electron micrographs of three potato flour samples are shown in Figure 1. Obviously, no starch granules were gelatinized in three PFs obtained by freeze-drying, ethanol-dehydration and oven drying at 40 °C, indicating the drying methods used were not destructive to starch granules. With three drying methods, the obviously pores and channels on the PF-FD samples (Figure 1, PF-FD). All the pores and channels on the PF-FD tests are likely caused by the constrained mutilation of potato starch granules due to local explosive release of water vapor from the built-up weight interior the unbending granules, as the water molecules attempt to elude through the strong inner structure and smooth surface structure beneath the vacuum conditions [5]. The ED (Figure 1, PF-ED) and OD (Figure 1, PF-OD) processes barely affected the surface microstructure of potato starch during the temperature range of 25–40 °C, except for rough surface due to the loss of free water.

### 3.3. X-ray Diffraction (XRD)

**Figure 2:**
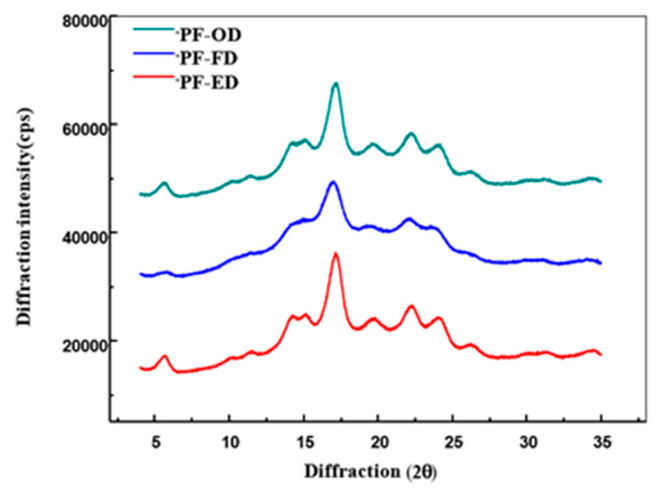
XRD patterns of potato flour samples subjected to different drying methods.PF-ED: potato flour dried for ethanol drying; PF-FD: potato flour dried for freeze drying; PF-OD: potato flour dried for oven drying.

Three potato flours presented different X-ray diffraction patterns (Figure 2). In comparison, the PFs obtained in this study showed typical B-type diffraction patterns of potato starch with peaks at 5.6, 17.0, 22.0 and 24.0° (2θ), indicating that starch granules were not disrupted upon drying of potato flour. Of the three drying methods, freeze-drying seemed to cause some disruption to crystallites of starch granules, as shown by the weaker diffraction peaks of PF-FD compared with those of PF-ED and PF-OD. The relative crystallinity of PF-FD was 31.3%, slightly lower than that of PF-ED (34.2%) and PF-OD (33.5%). Freeze-drying has been reported to disrupt the crystalline structure of B-type potato starch and C-type chestnut starch but have little effect on A-type maize starch. These results also indicated that ethanol drying and oven drying at 40 °C have little effect on crystallinity of potato starch.

### 3.4. LCM-Raman Spectroscopy

The FWHM values of the band at 480 cm^−1^ differed significantly between three PFs (Table 1). PF-FD had the biggest FWHM value of 16.8, followed by PF-OD sample of 14.9, PF-ED samples had the littlest FWHM value of 13.8. The ED process has the less impact on the short-range atomic arrange of starch within the potato flour. The freeze-dried potato flour appeared a better FWHM esteem compared with the comparing oven drying potato flour, demonstrative of the disturbance of the requested structure. 

### 3.5. Thermal Properties

The DSC thermograms and thermal transition temperatures (To, Tp, Tc) and enthalpy change (ΔH) of potato flours are shown in Table 2, PF-ED and PF-OD samples showed similar thermal transition temperatures and enthalpy changes, but PF-FD sample showed lower thermal transition temperatures and enthalpy changes. These observations were generally compliance with XRD and Raman results, suggesting that freeze drying damaged the long- and short-range molecular order of potato starch. The lower thermal transition temperatures of PF-FD sample indicated that more stable crystallites were disrupted, consistent with the thermally treated potato starch, which showed lower transition temperatures than untreated samples. This was explained as being due to the destruction of starch crystallites occurring preferentially at the outer surface of granules, which are more stable than those at the center of granules. 

### 3.6. Pasting Properties

**Figure 3:**
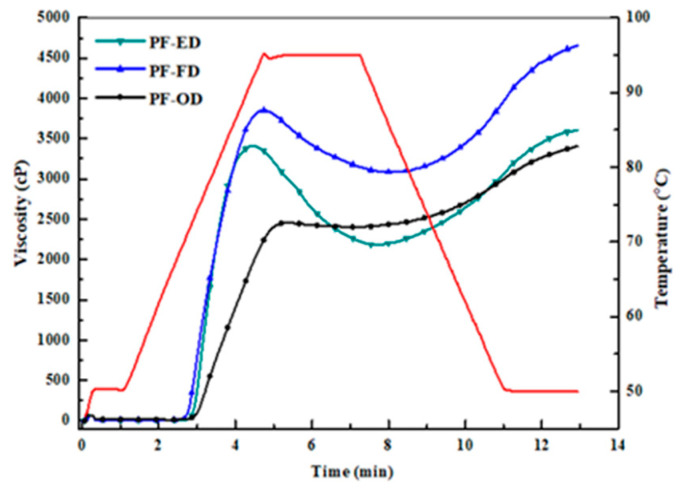
RVA profiles of potato flour samples subjected to different drying methods. PF-ED: potato flour dried for ethanol drying; PF-FD: potato flour dried for freeze drying; PF-OD: potato flour dried for oven drying.

The pasting properties of three potato flours determined using an RVA differed greatly (Table 3 and Figure 3). In contrast, the PF-FD and PF-ED samples showed the typical pasting profiles of potato starch, namely very high peak and final viscosities. PF-FD sample presented higher pasting viscosities than did PF-ED, presumably due to the disruption of surface structure causing greater swelling of starch granules in PF-FD sample. The PF-OD sample showed a different pasting profile compared with other PSs, such as lower peak viscosity and breakdown viscosity. The lower breakdown value may be attributed to the enhanced order of amorphous regions that prevented the disruption of starch granules [15].

### 3.7. Gel Textural Analysis

Gel textural properties of three potato flours were also different greatly (Table 4). PF-ED gel showed the highest hardness, gumminess and chewiness values, followed by PF-OD sample, and PF-FD sample presented the lowest values. The observation indicated that starch in PF-FD and PF-ED pastes retrograded to form a softer and more rigid gel network, respectively, than starch in PF-OD paste. The mechanical properties of starch gel depend on the volume division and rigidity of gelatinized starch granules, as well as the intelligent between scatter and persistent stage of gel [16]. These were influenced by different drying medications, coming about within the clear changes in gel texture.

### 3.8. Swelling Power and Starch Solubility

Swelling power and starch solubility of potato flour tests are displayed in Table 1. The PF-ED and PF-OD samples showed the similar values of swelling power and starch solubility, and the PF-FD showed the lower swelling power and starch solubility values. The higher swelling power and solubility of PFs are due to the potato starch have a higher content of phosphate groups on amylopectin, and repulsion between phosphate groups on adjacent chains will increase hydration by weakening the extent of bonding within the crystalline domain [17].

### 3.9. Preparation of the Fresh Potato Noodles

To determine the maximum addition of potato flour to wheat flour, we performed the pre-experiment, and the maximum addition of potato flour to wheat flour is 40% for PF-FD, and 50% for both PF-ED and PF-OD, the dough with more addition amount of PFs cannot be prepared the noodles. Previous studies showed that noodles with cooked whole potato flour content below 40% were acceptable [18], ethanol drying and oven drying enhance the addition amount of PFs in the wheat flour products.

### 3.10. Color of Potato Noodles

Color is considered as a major determinant of noodle quality. For white salt noodles, a clean and bright appearance is alluring, while dark or gray colors are negative traits. Color parameters of noodles are shown in Table 5. As the amount of potato flour increased in noodles, the L* (lightness) values of noodles decreased gradually, while the a* (redness) and the b* (yellowness) values increased, indicating the addition of PF made the noodles less bright compared with those without PF. 

### 3.11. Textural Profile Analysis (TPA) 

Table 6 shows the texture profile analysis (TPA) arguments of noodles with different PF/WF ratios. Hardness and springiness are of primary concern for consumers of noodle products. The hardness and springiness value of fresh potato noodles were increased with addition PF (dried by different drying method) content. Fresh potato noodles with ODPF have the largest hardness and springiness value.

## 4. Discussion

In this study, we investigated the impact of three drying methods on structural and functional properties of PFs and the quality characteristics of noodles prepared by different PF/WF ratio. As a result, the FD treatment led to pores and channels on the surface of potato starch and decreased the gelatinization temperatures. The damage of the crystalline arrangement and the diminishment within the amount of double helices might have happened amid the FD process [5]. A similar result was also observed with chestnut starches after freeze-drying [8]. The PF-FD samples have a higher peak viscosity compared with PF-ED and PF-OD samples. A conclusion is that the PF prepared by freeze drying have a stronger ability to compete for water due to the disruption of starch granules under the vacuum conditions. The PF-OD samples did not significantly the surface granular morphology, but the breakdown values and final viscosity show some differences. The decreased breakdown values may be attributed to the enhanced order of amorphous regions that prevented the disruption of starch granules [15]. The ED treatment has the least effect on the potato starches, but it obtained the most water in the potato flour due to the room temperature. Gelatinization temperature is a vital parameter in deciding potato starch application in nourishment. Therefore, the results have great significance for choosing the appropriate drying methods of potato flour and the development of potato staple product.

The quality characteristics of noodles by mixing different PF/WF ratio were investigated, the maximum addition of potato flour to wheat flour is 40% for PF-FD, and 50% for both PF-ED and PF-OD, the different values were attributed to the different drying methods that have significant effects on the surface granular morphology, long- and short-range requested structure of potato starches, the physicochemical characteristics of starch play a fundamental part in both noodle preparing and the ultimate noodle quality [19]. The greatest expansion of potato mash in steamed bread was 30% due to the dough tests with potato mash substance of more than 30% debilitated starch–gluten interaction and displayed spasmodic arrange structures [20]. The addition of PFs increased to the 40% and 50% attributed to the potato starch increased the dough extensibility [21], the structure and functional properties of starch granules can affect the content of potato flour in the wheat flour products. Previous study also pointed that the texture profile examination of noodles may be due to the interactions between the components of potato and wheat flours or the water assimilation in potato-wheat system [22]. Potato noodles were less brighter compared with wheat flour noodles, the color of steamed bread containing potato flour moreover darkened and inclined towards yellow and red compared with that of steamed bread containing as it were wheat [11]. The darkening and light-yellow color of steamed bread enhanced with potato mash may be credited to the browning caused by polyphenol oxidase in potato mash during prepared [20]. 

Fresh noodles with mixing wheat flour/potato flour are a complex system; potato starch content and the gluten protein network were the primary factor influencing the noodle’s texture properties. In common, pasting properties are related to the swelling and break of starch granules in a system [16]. PV is a pointer of ease with which the starch granule is deteriorated and frequently related with final product quality [23]. Zaidul [24] detailed that the PV of wheat-potato starch mixtures was higher than those of the wheat-sweet potato starch, wheat-yam starch, and wheat-cassava starch because of the higher phosphorus and lower amylose substance of potato starch, which come about in higher swelling of potato starch than that of sweet potato starch, yam starch and cassava starch. FV was detailed that filtered starch molecules may be mindful for the expanded FV. FV of flour was altogether (*p* < 0.001) related with buckwheat noodle hardness, which uncovered that high final viscosity accounted for rough and stiff noodles [25]. Setback, which is a measure of retrogradation propensity of the starch was altogether correlated with the hardness of fresh noodles. Among the three drying methods, the potato flour by oven drying (ODPF) has the highest value of PV, FV and SB and the fresh noodles with ODPF has the highest value of hardness and springiness. The ODPF has the largest impact on the quality characteristics of wheat flour noodles, these results are of great interest in the optimization of potato processing, and to manipulate the quality attributes of finalized food products.

## 5. Conclusions

This work deeply evaluated the effects of three drying methods on the structure and functional properties of potato flour and the quality properties of fresh potato noodles. The freeze-drying disrupted the surface and the structure of potato starch granules, compared with the ethanol drying and oven drying methods. ED and OD treatments had less effect on the surface and structure of potato starch, and it increased the potato flour content of noodles attain to 50%. The effects of different drying methods on potato starch granules determines the quality characteristics of potato noodles. This study provides valuable information for the selection of suitable drying methods of potato flour applied in the flour products and a theoretical basis for the application of potato products.

## Figures and Tables

**Table 1:** Chemical composition, XRD crystallinity, FWHM at 480 cm^−1^, swelling power and starch solubility of potato flours.

Samples	Moisture (%)	Total Starch Content (%)	XRD Crystallinity (%)	FWHM at 480 cm^−1^	Swelling Power (g/g)	Starch Solubility (%)
PF-ED	10.5 ± 0.2 ^c^	70.5 ± 0.9 ^a^	34.2	13.8 ± 0.3 ^a^	15.2 ± 0.2 ^b^	7.1 ± 0.6 ^b^
PF-FD	5.8 ± 0.1 ^a^	75.4 ± 0.5 ^c^	31.3	16.8 ± 0.4 ^c^	12.8 ± 0.0 ^a^	4.6 ± 0.2 ^a^
PF-OD	8.7 ± 0.3 ^b^	73.5 ± 0.2 ^b^	33.5	14.9 ± 0.5 ^b^	15.1 ± 0.2 ^b^	7.3 ± 0.8 ^b^

Values are means ± SD. Values with the different letters in a column are significantly different (*p* < 0.05).

**Table 2:** Thermal properties (measured by DSC) of potato flours.

Samples	T_o_ (°C)	T_p_ (°C)	T_c_ (°C)	ΔH (J/g)
PF-ED	64.6 ± 0.1 ^b^	68.4 ± 0.1 ^b^	73.6 ± 0.1 ^b^	14.5 ± 0.0 ^b^
PF-FD	61.5 ± 0.1 ^a^	66.8 ± 0.1 ^a^	72.5 ± 0.2 ^a^	13.4 ± 0.3 ^a^
PF-OD	65.0 ± 0.1 ^c^	68.8 ± 0.2 ^c^	73.7 ± 0.1 ^b^	14.4 ± 0.3 ^b^

Values are the means ± SD. Values with the different letters in a column are significantly different (*p* < 0.05). N.D., not detected.

**Table 3:** Pasting properties of potato flours.

Samples	PV (cP)	TV (cP)	BD (cP)	FV (cP)	SB (cP)	PT (°C)
PF-ED	3431.7 ± 22.5 ^b^	2181.7 ± 2.5 ^a^	1250.0 ± 25.0 ^c^	3556.0 ± 49.0 ^a^	1406.0 ± 15.0 ^b^	72.6 ± 0.1 ^b^
PF-FD	3821.0 ± 31.0 ^c^	3055.0 ± 31.0 ^c^	766.3 ± 0.6 ^b^	4608.7 ± 49.5 ^b^	1553.7 ± 18.5 ^c^	70.2 ± 0.1 ^a^
PF-OD	2516.7 ± 58.5 ^a^	2472.0 ± 67.0 ^b^	44.7 ± 8.5 ^a^	3548.7 ± 143.5 ^a^	1076.7 ± 76.5 ^a^	73.7 ± 0.3 ^c^

Values are the means ± SD. Values with the different letters in a column are significantly different (*p* < 0.05). N.D., not detected.

**Table 4:** Gel textural properties of potato flour by different drying methods.

Samples	Hardness (N)	Springiness	Cohesiveness	Gumminess	Chewiness	Resilience
PF-ED	525.3 ± 5.2 ^b^	0.86 ± 0.00 ^a^	0.82 ± 0.03 ^c^	430.1 ± 19.1 ^c^	371.2 ± 17.8 ^b^	0.61 ± 0.04 ^c^
PF-FD	373.0 ± 23.5 ^a^	0.95 ± 0.02 ^b^	0.78 ± 0.00 ^b^	290.9 ± 20.1 ^a^	276.4 ± 23.5 ^a^	0.56 ± 0.01 ^b^
PF-OD	495.9 ± 40.6 ^b^	0.85 ± 0.04 ^a^	0.69 ± 0.01 ^a^	343.1 ± 33.4 ^b^	293.2 ± 43.5 ^a^	0.45 ± 0.01 ^a^

Values are the means ± SD. Values with the different letters in a column are significantly different (*p* < 0.05). N.D., not determined.

**Table 5:** Color properties of fresh noodles.

Samples	L*	a*	b*
WFN	94.54	0.01	7.52
PFN-ED10%	91.87	0.27	7.27
PFN-ED20%	90.76	0.45	7.17
PFN-ED30%	89.07	0.63	7.62
PFN-ED40%	87.40	0.80	8.02
PFN-ED50%	86.22	0.94	8.51
PFN-FD10%	92.64	0.11	7.06
PFN-FD20%	91.23	0.21	7.04
PFN-FD30%	89.98	0.32	7.39
PFN-FD40%	89.11	0.43	7.46
PFN-OD10%	91.43	0.36	7.12
PFN-OD20%	89.28	0.68	7.46
PFN-OD30%	86.93	0.96	7.87
PFN-OD40%	85.24	1.17	8.51
PFN-OD50%	83.46	1.37	8.89

Values are the means ± SD. Values with the different letters in a column are significantly different (*p* < 0.05). WFN and PFN represent wheat flour noodles and potato flour noodles, respectively. PFN-x% represents potato flour noodles made of x% potato flour and (1 – x%) wheat flour. L*: lightness, a*: redness, b*: yellowness.

**Table 6:** Textural characteristics of fresh potato noodles.

Samples	Hardness (N)	Springiness	Cohesiveness	Gumminess	Chewiness	Resilience
WFN	7604.3 ± 68.1 ^a^	0.29 ± 0.01 ^a^	0.33 ± 0.01 ^d,e^	2697.6 ± 150.2 ^b,c^	780.7 ± 81.0 ^a^	0.25 ± 0.02 ^b,c^
PFN-EDPF10%	8675.4 ± 70.0 ^b^	0.30 ± 0.01 ^a^	0.30 ± 0.01 ^b^	2399.7 ± 246.0 ^a,b^	719.3 ± 74.5 ^a^	0.25 ± 0.01 ^b^
PFN-EDPF20%	9455.9 ± 9.9 ^c^	0.35 ± 0.01 ^c,d^	0.32 ± 0.01 ^c,d^	3186.6 ± 116.3 ^d,e^	1107.4 ± 80.0 ^b,c^	0.27 ± 0.01 ^c,d^
PFN-EDPF30%	11,601.7 ± 107.4 ^f^	0.38 ± 0.01 ^e^	0.34 ± 0.01 ^f,g^	3889.3 ± 194.4 ^f^	1503.5 ± 102.9 ^e^	0.29 ± 0.01 ^e,f^
PFN-EDPF40%	12,784.1 ± 193.2 ^g^	0.41 ± 0.01 ^f^	0.34 ± 0.00 ^f,g^	4348.6 ± 36.6 ^g^	1793.9 ± 20.7 ^f^	0.30 ± 0.01 ^f^
PFN-EDPF50%	15,325.6 ± 17.4 ^j^	0.46 ± 0.01 ^g^	0.36 ± 0.00 ^h^	5254.0 ± 274.9 ^i^	2388.2 ± 66.3 ^h^	0.33 ± 0.01 ^g^
PFN-FDPF10%	9867.0 ± 87.8 ^d^	0.30 ± 0.01 ^a^	0.31 ± 0.00 ^c^	2609.1 ± 448.5 ^b,c^	774.7 ± 149.2 ^a^	0.27 ± 0.00 ^d^
PFN-FDPF20%	10,805.6 ± 132.9 ^e^	0.32 ± 0.00 ^b^	0.32 ± 0.01 ^c,d^	3495.4 ± 4.6 ^e^	1123.6 ± 13.5 ^b,c^	0.28 ± 0.00 ^d^
PFN-FDPF30%	14,495.0 ± 36.0 ^i^	0.36 ± 0.02 ^d^	0.38 ± 0.01 ^i^	5678.0 ± 386.3 ^j^	2051.9 ± 90.1 ^g^	0.34 ± 0.01 ^g^
PFN-FDPF40%	15,737.9 ± 107.9 ^j^	0.43 ± 0.00 ^f^	0.38 ± 0.01 ^i^	6224.2 ± 234.8 ^k^	2645.4 ± 93.9 ^i^	0.36 ± 0.01 ^h^
PFN-ODPF10%	8517.4 ± 100.5 ^b^	0.30 ± 0.01 ^a^	0.29 ± 0.01 ^a,b^	2193.0 ± 189.6 ^a^	664.8 ± 81.3 ^a^	0.25 ± 0.01 ^b,c^
PFN-ODPF20%	9298.8 ± 17.3 ^c^	0.33 ± 0.00 ^b,c^	0.29 ± 0.01 ^a^	2364.6 ± 51.1 ^a,b^	790.7 ± 9.2 ^a^	0.23 ± 0.00 ^a^
PFN-ODPF30%	12,343.1 ± 179.6 ^g^	0.35 ± 0.01 ^c,d^	0.34 ± 0.00 ^f,g^	4143.6 ± 0.9 ^f,g^	1431.1 ± 21.3 ^d,e^	0.30 ± 0.00 ^e,f^
PFN-ODPF40%	13,591.0 ± 150.5 ^h^	0.49 ± 0.00 ^h^	0.35 ± 0.00 ^g^	4689.0 ± 7.6 ^h^	2536.5 ± 243.7 ^i^	0.30 ± 0.01 ^f^
PFN-ODPF50%	16,573.7 ± 301.9 ^k^	0.53 ± 0.02 ^i^	0.38 ± 0.00 ^i^	6379.3 ± 249.7 ^k^	3348.0 ± 6.8 ^j^	0.35 ± 0.01 ^h^

Values are the means ± SD. Values with the different letters in a column are significantly different (*p* < 0.05). WFN and PFN represent wheat flour noodles and potato flour noodles, respectively. PFN-x% represents potato flour noodles made of x% potato flour and (1 – x%) wheat flour.

## Data Availability

Not applicable.
